# G protein-coupled estrogen receptor 1 and collagen XVII endodomain expression in human cutaneous melanomas: can they serve as prognostic factors?

**DOI:** 10.3389/pore.2024.1611809

**Published:** 2024-08-26

**Authors:** Uğur Çakır, Petra Balogh, Anikó Ferenczik, Valentin Brodszky, Tibor Krenács, Sarolta Kárpáti, Miklós Sárdy, Péter Holló, Melinda Fábián

**Affiliations:** ^1^ Department of Dermatology, Venereology and Dermatooncology, Semmelweis University, Budapest, Hungary; ^2^ Queen Elizabeth Hospital, Cellular Pathology Department, University Hospitals Birmingham, Birmingham, United Kingdom; ^3^ Doctoral School of Economics, Business and Informatics, Corvinus University of Budapest, Budapest, Hungary; ^4^ Department of Health Policy, Institute of Social and Political Sciences, Corvinus University of Budapest, Budapest, Hungary; ^5^ 1st Department of Pathology and Experimental Cancer Research, Semmelweis University, Budapest, Hungary

**Keywords:** estrogen receptor, collagen xvii, prognostic factors, immunohistochemistry, prognosis

## Abstract

Melanoma incidence is increasing globally. Although novel therapies have improved the survival of primary melanoma patients over the past decade, the overall survival rate for metastatic melanoma remains low. In addition to traditional prognostic factors such as Breslow thickness, ulceration, and mitotic rate, novel genetic and molecular markers have been investigated. In our study, we analyzed the expression of G-protein coupled estrogen receptor 1 (GPER1) and the endodomain of collagen XVII (COL17) in relation to clinicopathological factors in primary cutaneous melanomas with known lymph node status in both sexes, using immunohistochemistry. We found, that GPER1 expression correlated with favorable clinicopathological factors, including lower Breslow thickness, lower mitotic rate and absence of ulceration. In contrast, COL17 expression was associated with poor prognostic features, such as higher tumor thickness, higher mitotic rate, presence of ulceration and presence of regression. Melanomas positive for both GPER1 and COL17 had significantly lower mean Breslow thickness and mitotic rate compared to cases positive for COL17 only. Our data indicate that GPER1 and COL17 proteins may be of potential prognostic value in primary cutaneous melanomas.

## Introduction

Melanoma is a life-threatening type of cancer that has shown a rising incidence worldwide over the past few decades [1, 2]. Major histopathological features predicting the prognosis of melanoma include Breslow thickness, ulceration, and mitotic rate [3]. In addition, age, gender, anatomical site, Clark level, lymphovascular and perineural invasion, regression, and tumor-infiltrating lymphocytes are important prognostic indicators and predictors of survival [3].

It has been observed that women exhibit a somewhat lower incidence and mortality rate of cutaneous melanoma, suggesting the role of sex hormones in tumorigenesis [4–6]. Consequently, estrogen receptors have been extensively investigated over the past decades in various malignancies, including melanoma [7–22]. Estrogen signaling and its effects are mediated through distinct receptor subtypes: ERα and ERβ, which are considered the classical estrogen receptors and function as ligand-activated transcription factors [23, 24]. Immunohistochemical and molecular studies have suggested that the downregulation of ERβ may serve as an indicator of metastatic potential in melanoma [25, 26]. Besides the classical estrogen receptors, estrogen signaling can also be mediated through the seven-transmembrane receptor, G-protein coupled estrogen receptor 1 (GPER1), which is expressed in various tissues under physiological conditions, including the nervous, reproductive, musculoskeletal systems, and gastrointestinal tract [27–30]. In tumor biology, GPER1 has been shown to facilitate cancer progression and migration, serving as a poor prognostic factor in ER-negative and human epidermal growth factor receptor 2 (HER2)-overexpressed breast and lung cancer cell lines [12–14, 19, 31, 32]. Conversely, it has also been associated with better overall survival in ER-positive and HER2-negative breast cancer [20, 32]. Furthermore, GPER1 has been reported to inhibit tumor proliferation in colorectal, prostate, and adrenocortical carcinomas, and GPER agonists have demonstrated growth inhibition in ovarian cancer [15–18, 22]. In the skin, GPER1 protein is thought to be essential for estrogen-mediated melanogenesis and is linked to hyperpigmentation during pregnancy through activation of the cAMP/pCREB/MITF signaling pathway, leading to melasma formation [33–35]. GPER1 activation inhibits melanoma proliferation in both human and murine melanoma cell lines and induces c-Myc depletion, which is associated with reduced PD-L1 expression, potentially enhancing the response to immune checkpoint inhibitor therapies [36, 37]. GPER1 knockdown has been shown to negate the effects of G-1 and tamoxifen, indicating a GPER1-dependent pathway and suggesting that GPER1 activation exerts anti-proliferative effects in melanoma [30].

In contrast to GPER1, which is associated with better outcome and mortality rates of cutaneous melanoma in women [4, 6], collagen XVII (COL17) appears to be implicated in skin cancer development [38, 39]. COL17 is a transmembrane glycoprotein of the hemidesmosome [40]. The intracellular domain of COL17 interacts with the intermediate filament network, while its extracellular domain anchors into the basement membrane zone, engaging with extracellular matrix proteins [41]. Beyond its structural role in the hemidesmosome complex, overexpression of COL17 is presumed to play a role in cancer development, tumor invasion, and decreased survival rates in squamous cell carcinoma, as well as colorectal, lung and pancreatic cancer [39, 42–47]. Contrarily, in human breast cancer, COL17 expression has been found to be decreased [48]. Krenács et al. showed that the cell-residual 60 kDa endodomain of COL17 (but not the shedding ectodomain) can be detected in primary and metastatic human melanoma as well as melanoma cell lines, but not in resting melanocytes and nevi [49].

As stated above, the existing literature has reported on the expression of GPER1 and COL17 in various tumors, including melanoma, using immunohistochemistry and mRNA levels, yet without specific emphasis on lymph node metastatic status. In our retrospective study, we aimed to investigate the expression of GPER1 and the cell-residual endodomain of COL17 proteins in primary melanoma samples with known lymph node status in relation to the major clinicopathological factors.

## Materials and methods

### Study population

Ethics approval was obtained from the Semmelweis University Institutional Review Board (32-4/2007). The database of the Department of Dermatology, Venereology and Dermatooncology of Semmelweis University was reviewed for melanoma patients who were diagnosed between 1st of January 2000, and 31st of December 2010, and completed a 10-year follow-up period. The total number of patients (n = 94) had an equal distribution of males (n = 47) and females (n = 47, respectively). The female patients were further subcategorized as premenopausal (<45 years old) and postmenopausal (≥45 years old) [50]. The registered clinicopathological characteristics are listed in Table 1. All examined melanoma samples were grouped according to the Breslow thickness categories reported in the 2018 American Joint Committee on Cancer (AJCC) melanoma staging system [51].

**TABLE 1:** Clinicopathological characteristics of the study population.

	Female	Male	Total
Total number of patients	47	47	94
Premenopausal	19 (40.42%)		
Postmenopausal	28 (59.58%)		
Anatomical site			
Trunk	16 (34.04%)	37 (78.72%)	53 (56.38%)
Upper extremities	7 (14.89%)	6 (12.77%)	13 (12.83%)
Lower extremities	24 (51.07%)	4 (8.51%)	28 (29.79%)
Melanoma subtype			
SSM	42 (89.36%)	41 (87.23%)	83 (88.30%)
NM	1 (2.13%)	4 (8.51%)	5 (5.32%)
LMM	1 (2.13%)	0 (0%)	1 (1.06%)
Unclassifiable	3 (6.38%)	2 (4.26%)	5 (5.32%)
Breslow thickness^a^			
≤1 mm	15 (31.91%)	18 (38.30%)	33 (35.11%)
>1, ≤2 mm	14 (29.79%)	8 (17.02%)	22 (23.40%)
>2, ≤4 mm	10 (21.28%)	10 (21.28%)	20 (21.28%)
>4 mm	8 (17.02%)	11 (23.40%)	19 (20.21%)
**Mean mitotic rate/HPF**	7.239 (±6.647)	6.851 (±6.438)	7.045 (±6.543)
Ulceration			
Present	14 (29.79%)	15 (31.91%)	29 (30.85%)
Absent	31 (65.96%)	29 (61.70%)	60 (63.83%)
No information	2 (4.25%)	3 (6.39%)	5 (5.32%)
Regression			
Present	11 (23.4%)	13 (27.66%)	19 (20.21%)
Absent	36 (76.60%)	34 (72.34%)	70 (74.47%)
Peritumoral lymphocytic infiltrate			
Present	28 (59.57%)	31 (65.96%)	59 (62.77%)
Absent	19 (40.43%)	15 (31.91%)	34 (36.17%)
No information	0 (0%)	1 (2.13%)	1 (1.06%)
***De-novo* melanoma**	40 (85.10%)	41 (87.23%)	81 (86.17%)
**Nevus-associated melanoma**	7 (14.89%)	6 (12.77%)	13 (13.83%)
Sentinel lymph node			
Positive	13 (27.66%)	13 (27.66%)	26 (27.66%)
Negative	34 (72.34%)	32 (68.09%)	66 (70.21%)
No information	0 (0%)	2^b^ (4.25%)	2^b^ (2.13%)
Distant lymph node metastasis			
Positive	4 (8.51%)	2 (4.26%)	6 (6.38%)
Negative	43 (91.49%)	45 (95.74%)	88 (93.62%)
Distant visceral/cutaneous metastasis (during the 10-year follow-up period)			
Detected	9 (19.15%)	4 (8.51%)	13 (13.83%)
Not detected	38 (80.85%)	43 (91.49%)	81 (86.17%)
**5-year survival**	45 (95.74%)	45 (95.74%)	90 (95.74%)
**10-year survival**	41 (87.23%)	44 (93.62%)	85 (90.43%)
**Mean follow-up time (months)**	139.545 (±33.879)	139.313 (±30.33)	139.429 (±32.105)

HPF, high-power field; LMM, lentigo maligna melanoma; NM, nodular melanoma; PLI, peritumoral lymphocytic infiltrate; SLNB, sentinel lymph node biopsy; SSM, superficial spreading melanoma.

^a^ Breslow thickness was categorized according to the 2018 American Joint Committee on Cancer (AJCC) melanoma staging system for all patients.

^b^ Instead of sentınel lymph node biopsy a block dissection was performed.

### Immunohistochemistry

The protein expressions of GPER1 and COL17 were evaluated in archived, formalin-fixed, paraffin-embedded melanoma tissue samples from primary melanomas of the 94 patients. Three µm thick sections were cut, dewaxed in xylene and rehydrated in decreasing concentrations of ethanol. Heat-induced epitope retrieval was done by boiling slides for 20 min in 500 mL of 0.1 M Tris-buffered-saline (TBS) containing 0.01 M ethylenediamine-tetraacetic acid (Tris-EDTA), pH 9.0, followed by 20 min cooling with open lid. The blocking of endogenous peroxidases was completed in 3% hydrogen peroxide (H_2_O_2_) diluted in methanol for 20 min, followed by blocking of non-specific antigen binding in TBS, pH 7.4, for 30 min containing 3% bovine serum albumin (BSA, #82-100-6, Millipore, Kankakee, Illinois, United States). For the detection of target proteins, rabbit polyclonal GPER1 (#NLS4271, 1:100; GPER/GPR30, Bio-Techne, Abingdon, United Kingdom) and mouse monoclonal anti-COL17 antibody clone 9G2, recognizing the aa507–529 uppermost extracellular part of COL17a endodomain region, as produced, validated and described by Stelkovics et al. [42, 49] antibodies were applied overnight in a humidified chamber. Tissue-bound antibody detection was performed with MACH4 Universal HRP-polymer, biotin-free detection (Biocare Medical, Concord, MA, United States). Aminoethyl-carbazole (AEC) H_2_O_2_ chromogen-substrate system dissolved in 0.1 M acetate buffer, pH 4.6, was used for revealing the peroxidase activity under microscopic control followed by nuclear staining using hematoxylin. After mounting, stained slides were digitalized using Panoramic Scan (3DHISTECH, Budapest, Hungary).

### Assessment of immunostaining

**FIGURE 1:**
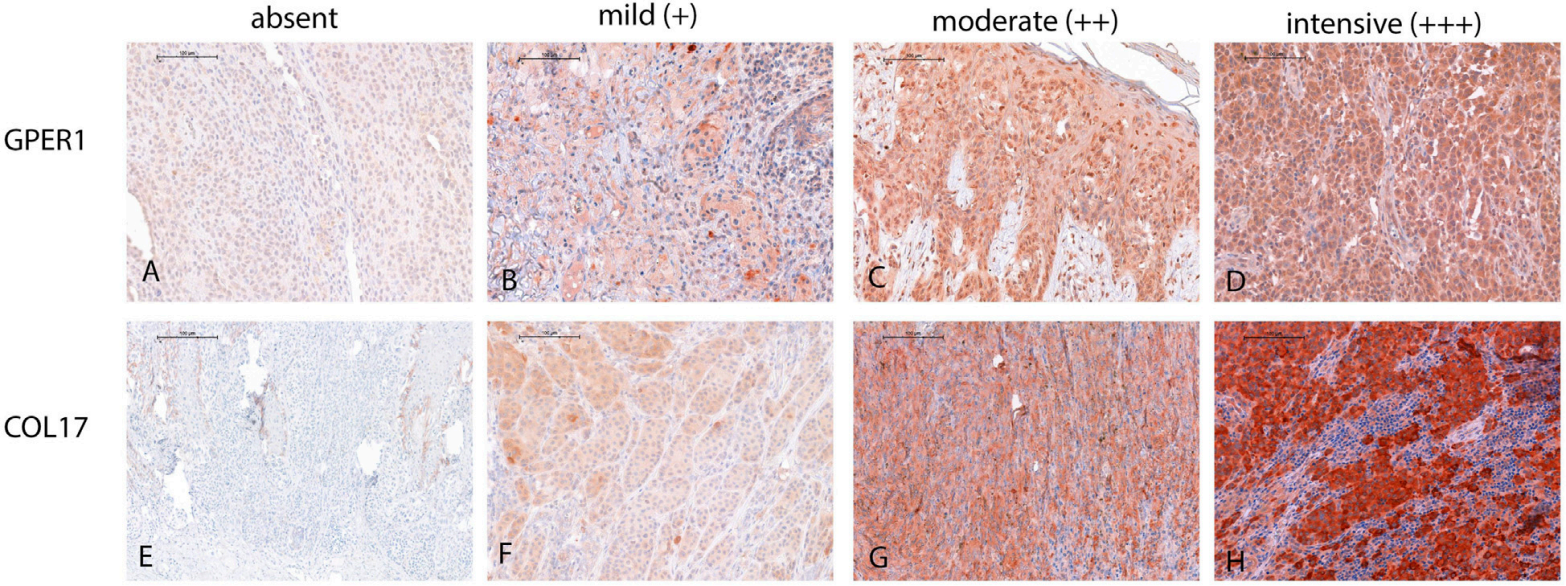
Illustration of GPER1 and COL17 protein expression intensity in the four-tier system in melanoma. **(A)** Representative image demonstrating the absence of GPER1 labelling. **(B–D)** Intensity differences between GPER1 positive tumor cells. **(E)** Absence of COL17 labelling of the tumor cells, only healthy keratinocytes at the basal layer showed positivity for COL17. **(F–H)** Intensity differences between COL17 positive tumor cells (for all images: original magnification ×40, scale bar: 100 µm).

Single immunolabeling for GPER1 and COL17 was completed on serial sections from each sample. Immunohistochemical scoring for both proteins was completed using the Histo-score (H-score), incorporating both the staining intensity and a percentage of stained tumor cells at each intensity level. The staining intensity values were indicated as 0 (absent), 1 (mild staining), 2 (moderate staining), and 3 (intensive staining) (Figure 1), which were then multiplied with the percentage of stained cells in the sample (ranging from 0% to 100%). The final H-score is derived from the sum of staining intensity values multiplied by percentage of stained cells as the equation shown. This score, therefore, is in the range of 0–300 (H-score = [0 × (% cells 0) + 1 × (% cells 1+) + 2 × (% cells 2+) + 3 × (% cells 3+)]) [52–55].

In order to minimize potential bias, the scoring was independently performed by four investigators (referred to as UC, MF, PB, TK). Disagreements in scoring were resolved through discussion and mutual consensus. Breast cancer tissue microarray (TMA) samples served as positive external controls for GPER1. As internal positive control of GPER1 staining, keratinocytes as well as sebaceous and sweat gland epithelia were used. For COL17, the surrounding healthy skin in each sample was deemed as positive control. Omitting the primary antibodies in each run served as negative controls.

### Statistical analysis

First, descriptive statistics were calculated for all variables. Then bivariate analyses were conducted, non-parametric Mann-Whitney U test was performed between groups and Pearson correlation analysis was used to determine the strength of the association between continuous variables, while cross tabulation analysis with Spearman’s chi-squared tests was performed between categorical variables. Statistical analyses were carried out by IBM SPSS Statistics for Windows, Version 25.0. Armonk, NY: IBM Corp.

## Results

### GPER1 protein expression and clinicopathological factors of melanoma

GPER1 expression was detected in over half of the melanoma samples (n = 54/94, 57.45%). The majority of thinner melanomas [Breslow thickness ≤2 mm (n = 40/57, 70.18%)] were positive for GPER1.

**FIGURE 2:**
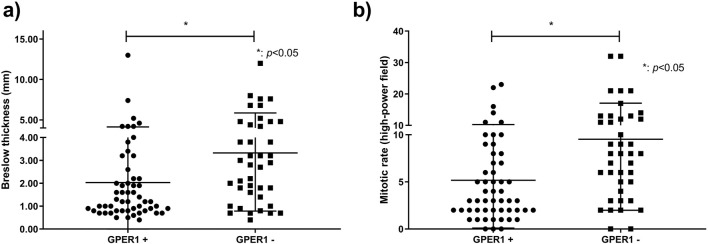
Association of Breslow thickness and mitotic rate with GPER1 protein expression. Dots and squares represent Breslow thickness (n = 94) **(A)** and mitotic rate (n = 93) **(B)** of individual primary melanomas.

GPER1 positive melanoma samples had significantly lower Breslow thickness (*p* = 0.01) and mitotic rate (*p* = 0.007) when compared to GPER1 negative cases (Figure 2). Mean H-score of GPER1 positive cases with Breslow thickness of more than 2 mm (H-score: 47.69) was significantly lower when compared with melanoma samples with Breslow thickness of 2 mm and less (H-score: 94.90) (*p* < 0.005). Furthermore, the presence of GPER1 receptor positivity showed inverse correlation with the presence of ulceration and sentinel lymph node (SLN) positivity based on cross-tabulation analyses (Table 2).

**TABLE 2:** Association of GPER1 protein expression with clinicopathological factors.

		GPER1		
		Negative	Positive	*p*-value
Ulceration	Absent	20	40	0.004
	Present	19	10	
Sentinel lymph node	Negative	25	41	0.084
	Positive	15	11	
Regression	Absent	31	39	0.562
	Present	9	15	

Numbers represent case counts.

With regards to the different subtypes of melanoma, GPER1 protein was expressed in the majority of superficial spreading melanomas (SSM, n = 50/83, 60.20%), while it was only seen in one case out of the five nodular melanomas (NM) (Supplementary Table S1).

Considering the gender difference, GPER1 protein expression was more frequently detected in females compared to males (n = 31/47 versus n = 23/47, *p* = 0.095) (Supplementary Table S1).

GPER1 expression showed no statistically significant relationship with peritumoral lymphocytic infiltrate (PLI), lymph node or distant metastases, patient’s age or between pre- and postmenopausal women group (Supplementary Table S1).

### COL17 endodomain expression and clinicopathological factors of melanoma

**FIGURE 3:**
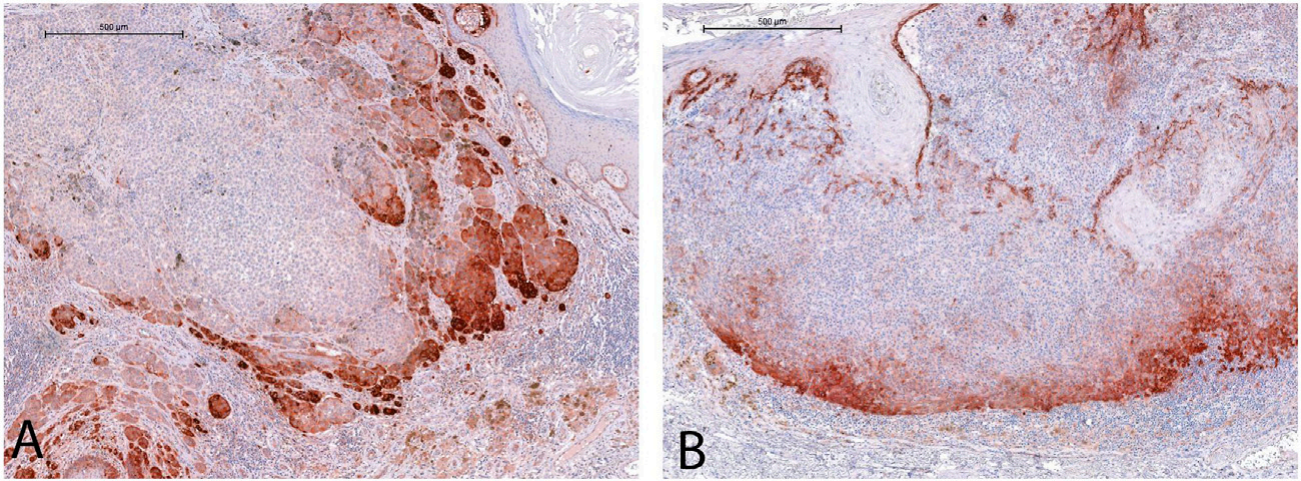
Cytoplasmic COL17 protein expression was mostly observed along the lateral **(A)** and deep **(B)**, growing fronts of the tumors. [See endogenous positive control reaction in the basal epidermal layer in **(B)**] (For both images: original magnification ×5, scale bar: 500 µm).

COL17 endodomain expression was found in nearly two-third of the melanoma samples (n = 62/94, 65.96%), and was predominantly present along the growing tumor front (Figure 3). The majority of thicker melanomas [Breslow thickness >2 mm (n = 32/37, 86.48%)] were positive for COL17 endodomain.

**FIGURE 4:**
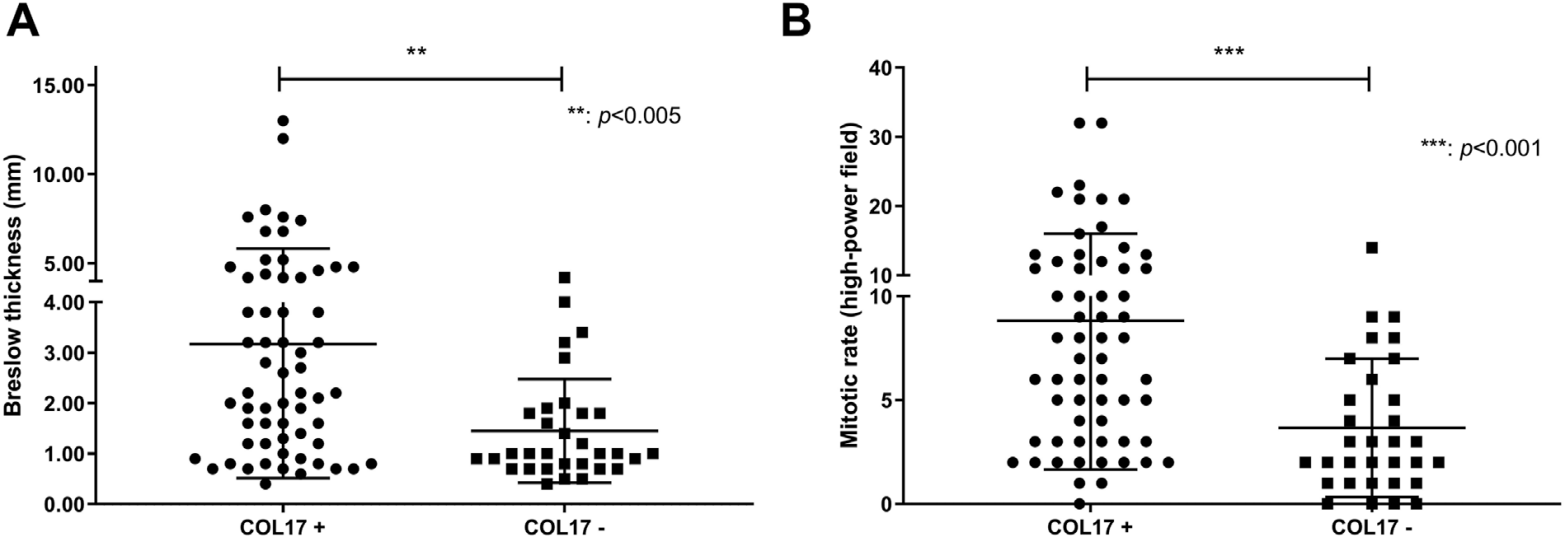
Association of Breslow thickness and mitotic rate with COL17 protein expression. Dots and squares represent Breslow thickness (n = 94) **(A)** and mitotic rate (n = 93) **(B)** of individual primary melanomas.

COL17 endodomain expression showed positive correlation with increased Breslow thickness (*p* = 0.004) and mitotic rate (*p* = 0.0009) (Figure 4). Mean H-score of COL17 endodomain positive cases with Breslow thickness of more than 2 mm (H-score: 77.44) was significantly higher than cases with Breslow thickness of 2 mm and less (H-score: 29.81) (*p* < 0.001). Furthermore, COL17 endodomain-expressing tumors exhibited more frequently unfavorable characteristics such as ulceration, regression, and SLN positivity (Table 3).

**TABLE 3:** Association of COL17 protein expression with clinicopathological factors.

		COL17		
		Negative	Positive	*p*-value
Ulceration	Absent	28	30	<0.0001
	Present	3	26	
Sentinel lymph node	Negative	27	38	0.056
	Positive	5	20	
Regression	Absent	28	41	0.043
	Present	4	19	

Numbers represent case counts.

The presence of distant lymph node and cutaneous/visceral metastases was more commonly seen in samples with COL17 endodomain expression, compared to COL17 negative tumors (Supplementary Table S2). The expression of the protein was observed more frequently in melanomas located on the trunk (n = 37/53, 69.81%) and upper extremities (n = 10/13, 76.92%) than in those with localization along the lower extremities (n = 13/28, 46.43%) (Supplementary Table S2).

Considering the histological subtypes, almost all nodular melanoma cases (n = 4/5, 80.00%) showed COL17 endodomain expression, and a subset of superficial spreading melanomas were also positive for COL17 endodomain (54/83, 65.00%) (Supplementary Table S2).

COL17 endodomain expression showed no significant association with gender, age, or presence of PLI (Supplementary Table S2).

### GPER1 and COL17 double expression

Nearly one-third (n = 32/94, 34.04%) of the cases exhibited simultaneous positivity for GPER1 and COL17 proteins. Melanomas positive for both markers had significantly lower mean Breslow thickness (2.425 mm vs. 3.957 mm, *p* = 0.004) and mitotic rate (6.61/HPF vs. 11.1/HPF, *p* = 0.008) compared to cases positive for COL17 only (Table 4). Conversely, double-positive cases had higher mean Breslow thickness (2.425 mm vs. 1.464 mm, *p* = 0.056) and mitotic rate (6.61/HPF vs. 3.14/HPF, *p* = 0.003) compared to cases positive for GPER1 only (Table 4).

**TABLE 4:** Comparison of mean Breslow thickness and mean mitotic rate of GPER1/COL17 double positive cases (n = 32) with GPER1 (n = 22) or COL17 (n = 30) positive cases.

	Mean Breslow thickness	Mean mitotic rate		Mean Breslow thickness	Mean mitotic rate
GPER1 positive	1.464 mm	3.14/HPF	COL17 positive	3.957 mm	11.1/HPF
GPER1/COL17 double positive	2.425 mm	6.61/HPF	GPER1/COL17 double positive	2.425 mm	6.61/HPF
	*p* = 0.056	*p* = 0.003		*p* = 0.004	*p* = 0.008

HPF, high-power field.

## Discussion

In this retrospective study, we aimed to explore the expression patterns of GPER1 and the COL17 endodomain proteins in primary cutaneous melanoma tissue samples from patients with known sentinel lymph node status and correlated this with the common clinicopathological features of melanoma.

GPER1 protein expression has been described in various tissues under physiological conditions including the nervous-, reproductive-, musculoskeletal system, and gastrointestinal tract [27–29]. Human and animal studies both revealed the role of GPER1 in the regulation of physiological responses involving mammary gland development [56], oocyte maturation [57, 58], endometrial cell growth [59], cardiomyocyte growth [60], vasodilation [61, 62], T-cell differentiation [63], inhibition of inflammation [64, 65], insulin secretion [61, 62], and chondrocyte differentiation [66]. In the murine skin, GPER1 mediates melanocyte differentiation and melanin pigment production; and it has also been shown to decreases expression of the oncodriver c-Myc, as described by Natale et al. using murine melanoma xenograft models [37].

Besides its expression in normal tissues, GPER1 has also been found in many types of cancers/cancer cell lines. Its role has been most intensively studied in breast cancer, where it appears to contribute to cancer cell proliferation, migration and invasion through its ability to transactivate epidermal growth factor receptors (EGFRs) [14, 67]. However, it was also shown to be a predictor of better overall survival (OS) in ER-positive breast cancer [20]. In ovarian cancer cells, GPER1 can be detected both within the nucleus and the cytoplasm, and the nuclear GPER1 is a potential predictive factor of poor survival [68]. Similar to its effect in breast cancer, GPER1 promotes cell proliferation, migration, lymph node metastasis and invasion in ER-negative ovarian cancer, and its overexpression correlates with tumor size and stage [69–71]. Cytoplasmic GPER1 overexpression was also found in high-grade estrogen receptor- and progesterone receptor-negative endometrial adenocarcinomas in association with myometrial invasion and poor survival [72]. Furthermore, GPER1 agonists enhance tumor growth of endometrial cancer cell line xenografts [73]. Similar to gynecological cancers, GPER1 (over) expression in lung cancer is also associated with high TNM stage and lymph node metastasis [13, 15, 74]. In human non-small-cell lung cancer cell lines, the expression of cytoplasmic GPER1 was high [13] and treatment of non-small-cell lung cancer cell lines with GPER1 antagonists impaired tumor growth [75]. In colorectal cancer, on the other hand, GPER1 appears to act as a tumor suppressor and its expression is negatively correlated with increased tumor stage and lymph node metastasis [16]. Moreover, higher GPER1 expression meant survival benefit for colorectal cancer patients [16]. In adrenocortical cancer, GPER1 presented tumor suppressive properties as GPER1 agonists suppressed adrenocortical carcinoma proliferation via cell cycle arrest, DNA damage, and apoptosis via ERK1/2 activation [18].

In our previous study [6], we found that GPER1 expression in pregnancy-related melanoma samples was associated with lower Breslow thickness, lower mitotic rate, lower hazard of local or distant metastases, and the protein expression was inversely associated with the presence of ulceration [6].

Our current study cohort, which included both genders and known sentinel lymph node status for all patients, showed similar results. GPER1 protein expression was correlated with lower Breslow thickness and mitotic rate. The presence of ulceration and sentinel lymph node metastases were found less frequently in GPER1-positive cases, suggesting that GPER1 may serve as a favorable prognostic marker.

Considering the role of GPER1 in cancer pathophysiology, it has been suggested as a potential target for cancer therapy [30]. *In vivo* and *in vitro* studies using GPER1 agonists such as G-1 demonstrated beneficial effects for cancer prognosis; these involved the inhibition of proliferation and the promotion of apoptosis in leukemia cell lines of T lineage [76], as well as the inhibition of growth in both mantle cell lymphoma [77] and pancreatic ductal adenocarcinoma [78]. Furthermore, G-1 assisted temozolamide to impair glioblastoma proliferation [79], and inhibited the growth of ovarian cancer cells [80] and gastric cancer [81]. In melanoma, the activation of GPER1 by its specific agonist G-1, inhibited the proliferation of mouse melanoma cell lines by decreasing cell division and blocking cell cycle progression in the G2 phase [36]. In a murine model, co-treatment with PD-1 inhibitor and GPER1 agonist G-1 resulted in reduced cell proliferation and tumor volume along with better survival [37]. *In vitro* treatment of uveal melanoma cell lines with GPER1 agonist induced mitotic arrest and apoptosis of tumor cells [82]. Based on these observations, an ongoing phase I clinical trial has been initiated using pembrolizumab and a selective agonist of GPER1 for treatment of melanoma [83].

After establishing the favorable prognostic aspects of GPER1, we sought to identify a marker that could potentially have reverse effects in our cohort. Based on our research group’s previous findings, namely, that uppermost extracellular part (aa507-529) of COL17a endodomain (but not the shedding ectodomain) is expressed in proliferating melanocytes and melanomas but not in benign melanocytic lesions [49], we aimed to examine COL17 expression in our current primary cutaneous melanoma cohort. The overexpression of COL17 coding mRNA and protein levels in colorectal carcinoma was associated with higher TNM staging, infiltrative growth, metastases, and decreased survival rates [45]. In lung cancer, elevated expression of COL17 was seen in the stromal environment and was associated with increased metastatic potential [47]. Furthermore, COL17 may contribute to the epithelial-to-mesenchymal transition and poor disease prognosis [84]. Overexpression of COL17 in cervix carcinoma exhibited a relation to increased local dissemination and metastasis [85, 86]. In contrast, COL17 showed reduced expression in advanced breast cancer [48] in correlation with higher TNM staging, increased invasion, postmenopausal status and poorer prognosis [48]. The antagonistic expression of COL17 in breast cancer is suggested to be due to hypermethylation of the COL17 coding gene promoter [86]. The role of COL17 was widely studied in both normal skin and cutaneous malignancies [39, 42, 43], also in head and neck squamous cell carcinoma (SCC) [87, 88]. SCC demonstrated COL17 overexpression promoting tumor invasion [42, 89]. Krenács et al. found that the cell residual endodomain, COL17, but not the ectodomain, was expressed in melanoma, but not in normal melanocytic and dysplastic nevi. It was also shown that immunological targeting of this protein sequence of COL17 with a specific antibody lead to apoptosis in melanoma cell lines [49]. Another study using murine melanoma models demonstrated that dysfunction of COL17 in keratinocytes promoted melanoma progression [90]. A recent publication demonstrated that in mucosal melanoma, the COL17 coding gene has a variant (p.Ser1029Ala) in the ectodomain, which may be less efficiently shed compared to the wild-type, thereby assisting melanoma progression [91]. All these previous data suggested the dysfunction of COL17 and its potential role in melanoma development and progression.

The current study shows that in primary melanoma samples, the expression of COL17 endodomain had an inverse relationship when compared to GPER1 expression by showing positive association with poor prognostic factors including increased Breslow thickness, higher mitotic rate and presence of ulceration. COL17 endodomain expression was more frequently seen in sentinel lymph node positive melanomas. Furthermore, the overexpression of COL17 along the invasive tumor front was suggestive of its potential role in tumor invasion in line with some earlier results: for example, COL17 was found to be at the invasive tumor fronts in squamous cell carcinoma of the tongue [89].

While there has been no direct interactions described so far between GPER1 and COL17 at a molecular level, COL17 protein has already been shown to inhibit breast cancer cell proliferation and growth via decreasing the phosphorylation of key proteins in the AKT/mTOR pathway [92], which is known to be one of the major downstream signaling target of the GPER1 pathway as well [93–96]. GPER1, in reverse, is found to enhance the phosphorylation of AKT/mTOR pathway leading to its activation in breast, ovarian and lung cancer resulting in increased cell proliferation in these types of cancers [93–95].

The rationale for investigating both GPER1 and COL17 concurrently was to determine whether their co-expression shows any correlation with the major clinicopathological factors of melanoma. Co-expression of both proteins in our study was observed in nearly one-third of the cases. Melanomas positive for both GPER1 and COL17 exhibited lower mean Breslow thickness and mitotic rates compared to cases positive for COL17 alone. Conversely, double-positive cases demonstrated higher mean Breslow thickness and mitotic rates than cases positive for GPER1 alone. Theoretically, while GPER1 expression decreases with increasing tumor size, COL17 expression emerges in high-risk, thicker melanomas, particularly at deeper invasive fronts. Our current results suggest that double positivity for GPER1 and COL17 may indicate an intermediate phase in tumor progression, however, future studies with larger cohorts and detailed molecular analyses are necessary to study any potential link between the two proteins and their potential interactions.

Although the association of GPER1 with favorable prognostic clinicopathological features suggests a trend towards better overall survival (OS), and COL17 endodomain is anticipated to correlate with poorer OS, a larger cohort of patients will be necessary in future studies to substantiate these findings.

In summary, our study demonstrated that GPER1 expression is associated with lower Breslow thickness, lower mitotic rate, absence of ulceration, and absence of sentinel lymph node metastasis in both genders, all of which are predictive clinicopathological factors for better survival outcomes. In contrast, COL17 endodomain expression in human melanoma, when present along the growing tumor front, suggests its involvement in melanoma invasion. COL17 endodomain expression was proven to be associated with poor prognostic features such as greater tumor thickness, higher mitotic rate, presence of regression, and sentinel lymph node positivity, indicating worse survival outcomes. Our findings suggest that the immunohistochemical detection of GPER1 and COL17 proteins in melanoma may serve as valuable prognostic markers.

Limitations of our study include the relatively small patient cohort, the retrospective study design, and the exclusive use of immunohistochemistry for biomarker detection. Further investigations with larger patient cohorts, supplemented by functional experiments, are necessary to corroborate our findings and elucidate the potential role of GPER1 and COL17 protein expression in melanoma.

## Data Availability

The raw data supporting the conclusions of this article will be made available by the authors, without undue reservation.
