# Supplementary material for: G protein-coupled estrogen receptor 1 and collagen XVII endodomain expression in human cutaneous melanomas: can they serve as prognostic factors?
Source: Pathol Oncol Res. 2024 Aug 26;30:1611809. doi: 10.3389/pore.2024.1611809 (PMC11381273; doi:10.3389/pore.2024.1611809)
Supplement: Supplementary file 1 [file DataSheet1.docx]

**Supplementary table 1.** Association of GPER1 protein expression with clinicopathological factors. Numbers represent case counts. *SSM: superficial spreading melanoma.* nodular melanoma, lentigo maligna melanoma, unclassifiable melanoma*

|  | | **GPER1** | |  |
| --- | --- | --- | --- | --- |
|  |  | **Negative** | **Positive** | ***p* value** |
| **Peritumoral lymphocytic infiltrate** | **Absent** | 13 | 21 | 0.583 |
|  | **Present** | 26 | 33 |  |
| **Distant lymph node metastasis** | **Negative** | 37 | 51 | 0.703 |
|  | **Positive** | 3 | 3 |  |
| **Distant visceral/cutaneous metastasis** | **Absent** | 34 | 47 | 0.777 |
|  | **Present** | 6 | 7 |  |
| **Gender** | **Male** | 24 | 23 | 0.095 |
|  | **Female** | 16 | 31 |  |
| **Menopausal status in case of female gender** | **Premenopausal** | 7 | 12 | 0.739 |
|  | **Postmenopausal** | 9 | 19 |  |
| **Melanoma subtype** | **SSM** | 33 | 50 | 0.132 |
|  | **All other*** | 7 | 4 |  |
| **Anatomical site** | **Lower extremity** | 8 | 20 | 0.146 |
|  | **Upper extremity** | 5 | 8 |  |
|  | **Trunk** | 27 | 26 |  |

**Supplementary table 2.** Association of COL17 protein expression with clinicopathological factors. Numbers represent case counts. *SSM: superficial spreading melanoma. * nodular melanoma, lentigo maligna melanoma, unclassifiable melanoma*

|  | | **COL17** | |  |
| --- | --- | --- | --- | --- |
|  |  | **Negative** | **Positive** | ***p* value** |
| **Peritumoral lymphocytic infiltrate** | **Absent** | 12 | 22 | 0.849 |
|  | **Present** | 19 | 38 |  |
| **Distant lymph node metastasis** | **Negative** | 31 | 55 | 0.335 |
|  | **Positive** | 1 | 5 |  |
| **Distant visceral/cutaneous metastasis** | **Absent** | 29 | 50 | 0.339 |
|  | **Present** | 3 | 10 |  |
| **Gender** | **Male** | 15 | 32 | 0.555 |
|  | **Female** | 17 | 28 |  |
| **Menopausal status in case of female gender** | **Premenopausal** | 7 | 11 | 0.900 |
|  | **Postmenopausal** | 10 | 17 |  |
| **Melanoma subtype** | **SSM** | 29 | 52 | 0.577 |
|  | **All other*** | 3 | 8 |  |
| **Anatomical site** | **Lower extremity** | 14 | 13 | 0.80 |
|  | **Upper extremity** | 3 | 10 |  |
|  | **Trunk** | 15 | 37 |  |
